# Partnership status and positive DNA methylation age acceleration across the adult lifespan in the UK

**DOI:** 10.1016/j.ssmph.2023.101551

**Published:** 2023-11-05

**Authors:** Wen Wang, Anna Dearman, Yanchun Bao, Meena Kumari

**Affiliations:** a Institute for Social and Economic Research, University of Essex, Wivenhoe Park, Colchester, Essex, CO4 3SQ, UK; b Department of Mathematics, University of Essex, Wivenhoe Park, Colchester, Essex, UK

**Keywords:** Partnership status, DNA methylation, Phenoage, DunedinPACE, Sex, Age, United Kingdom

## Abstract

Although a significant body of research has shown that married people are healthier and live longer, empirical research on sex differences in the link between marital status and health suggests results are mixed. Moreover, the sex disparities in marital status and health relationships vary across adulthood. The literature on partnership status and measures of ageing is largely focused on older age groups and is limited in its view of early adulthood. Data from waves 2 and 3 (2010–2012) of Understanding Society: UKHLS were used to examine the association of current partnership status with epigenetic age acceleration (AA) assessed with DNA methylation (DNAm) algorithms 'Phenoage' and ' DunedinPACE ' in 3492 participants (aged 16–97). Regression models were estimated separately for men and women, and further stratified by age groups.

Divorced/separated and widowed people showed positive age acceleration compared to the married/cohabiting people (reference group). Some sex differences were apparent, especially, among the single and divorced/separated groups. Age differences were also apparent, for example in men, being single was negatively associated with DNAmAA in the youngest group, but positively in the oldest group compared to partnered counterparts.

These findings illustrate the importance of partnerships on the ageing process, in particular marital change through divorce and widowhood for positive age acceleration in adults. For single groups, observations were heterogenous by age and sex.

## Highlights

• Slower epigenetic ageing is observed in men and women in partnerships.
• Divorced, separated, or widowed status is associated with faster epigenetic ageing.
• Singledom and its association with epigenetic ageing is moderated by sex and age.
• Results highlight the importance of findings from the entire adult age range.

## 1 Introduction

A significant body of research has shown that married people are healthier (both mentally and physically) and live longer compared to their unmarried (single, divorced, or widowed) counterparts (Bulanda et al., 2016; Carr & Springer, 2010; Hughes & Waite, 2009; Liu et al., 2020; McFarland et al., 2013; Tatangelo et al., 2017). Marriage or being in a partnership may be protective through the accumulation and sharing of economic, behavioural, and psychosocial resources between partners. However, health behaviours and socioeconomic position may influence the association between partnership status and health. Being in a partnership is beneficial for men and women, independent of a variety of confounders (Brenn & Ytterstad, 2016) with some studies suggesting a greater benefit in women than men (Wang et al., 2020). Associations of partnership status and health can be disease specific (Wang et al., 2020) and with broader health statuses such as frailty suggesting associations that are not system specific (Kojima et al., 2020). Marital disruption such as divorce may have negative impacts associated with stress and serve to undermine health (Crowley, 2019; Liu & Waite, 2014; McFarland et al., 2013). Further, the death of a spouse is associated with a short term increased risk of mortality (Ennis & Majid, 2021; Moon et al., 2011). As with partnership status, these associations may be moderated by factors such as sex and age, with different associations in different age groups (McFarland et al., 2013; Wang et al., 2020; Yu, 2023).

Explanations for the link between partnership status and health and wellbeing have centred on the marital resource model and the stress model by most researchers.

The marital resource model proposes an increase in health-enhancing resources such as economic, psychosocial, and social support associated with partnership (Waite & Gallagher, 2001). For instance, marriage or cohabitation leads to an increase in income and wealth due to specialization, economies of scale, and the pooling of resources within the household. Moreover, marriage/cohabitation is associated with an increase in psychosocial resources compared to divorce and widowhood, such as receiving support from partners and stronger ties with relatives, neighbours, and shared friends (Kalmijn, 2003, 2012). The accumulation of resource advantages enhances the opportunities to access resources that promote health, such as nutrition, high quality living environment, healthcare, and enable healthy behaviours to be maintained or unhealthy behaviours to be corrected.

The stress model puts more emphasis on relationship disruption, positing that divorce and widowhood may cause deleterious effects of short-term stress and sustained chronic strains, which undermine people's health (Umberson & Thomeer, 2020; Williams & Umberson, 2004). On the one hand, experiencing marital disruption may lead to grief, loss of support, changes in housing and daily life, and a decline in socio-economic position (SEP). Disadvantaged SEP reduces opportunities to access resources while increasing exposure to harmful stressors (Schreier & Chen, 2013). Response to stress may play a role in deteriorating health through various direct or indirect mechanisms. For example, response to stress can alter DNA methylation and cortisol sensitivity (Palma-Gudiel et al., 2015; Schiele et al., 2020), and sustained chronic strains may result in persistent epigenetic changes (Zhang & Liu, 2022), which may affect the ageing process and increase disease risks. Experiencing stressful events may make individuals more likely to adopt unhealthy behaviours (e.g., smoking and drinking) as a way of coping, and heavy drinking and smoking are directly linked to cellular ageing (Martins de Carvalho et al., 2019; Topiwala et al., 2022).

A number of studies suggest that the impact of relationship dissolution on a number of measures of health is different in men and women (Leopold, 2018; Percheski & Meyer, 2018; Zhang et al., 2021). There are observed patterns in sex differences in social network composition, such that men typically receive more emotional support and favourable regulation of health behaviours from family than women do, thus the impact of relationship disruption on men's health is greater than on women (Carr & Springer, 2010; McKenzie et al., 2018; Williams & Umberson, 2004). However, the sex disparities in marital status and health are moderated by age. Since the normative period for some social roles (e.g., marriage) occurs earlier for women than for men (Average age at marriage England and Wales, by gender | Statista), the psychological and physiological disadvantage from the accumulation of marital stress may be greater for women than men. In addition, societal changes in patterns of partnership over time leads to different potential associations of partnership with health which is different for different age groups. For example, as the average age of first marriages rose from 27.4 to 24.7 in 1972 to 39.7 and 37.3 by 2019 for men and women respectively (Average age at marriage England and Wales, by gender | Statista).

Previous research suggests that markers that reflect multiphysiological systems vary by partnership status, dissolution and widowhood (Kiecolt-Glaser, 2018; Kojima et al., 2020; Rote, 2017). At the molecular level, evidence suggests an association of partnership status and biomarkers of ageing such as telomere length and attrition (Chen et al., 2020; Whisman et al., 2016; Yu & Liu, 2021). Thus, there is potential to explore alternate molecular measures of ageing. Novel DNA methylation algorithms of ageing have been developed as a new method to encapsulate age related change experienced by individuals.

Ageing is an inevitable process experienced by all organisms, occurring at molecular, cellular, organ, and organismal levels. Over the course of biological ageing, there is compromised functionality and a decline in the reparative and regenerative capacities of tissues and organs. Such progressive loss of physiological integrity serves as the risk factor for major human pathologies (López-Otín et al., 2013). Great efforts have been undertaken to classify the molecular hallmarks of ageing, which have a vital role in both the ageing process and age-related diseases. In addition to telomere length, biological ageing and rapid ageing (expressed as “age acceleration” in this study), often calculated as a greater biological than chronological age, can be calculated using alternate methods, for example measurements have been developed using DNA methylation (DNAm) (Bell et al., 2019), which is the most widely studied epigenetic modification in population health. DNAm focuses on exploring cytosine methylation in CpG dinucleotides (CpG methylation). Initially, DNAm age algorithms were built by identifying DNAm patterns associated with chronological age. Recently, DNAm age algorithms, for example, the ‘Phenoage’ (Levine et al., 2018), ‘DunedinPoAm’ (Belsky et al., 2020), and ‘DunedinPACE’ (Belsky et al., 2022) algorithms have been described. For example, the ‘Phenoage’ DNAm algorithm (Levine et al., 2018) used clinical biomarkers and chronological age from the third National Health and Nutrition Examination Survey (NHANES) to predict a composite biological age score (so-called "phenotypic age"). Then they used elastic net regression and identified 513 CpG sites to predict the previously created phenotypic age. ‘DunedinPACE’ DNAm algorithm (Belsky et al., 2020) tracked within-individual decline over two decades in 19 multi organ system biological indicators as the predicted phenotype, then also used elastic net regression to identify CpG sites. These algorithms are trained using chronological age as well as phenotypic ageing measures that consist of clinical biomarkers, and are considered to represent the speed of biological age. Greater speed of ageing measured with these algorithms is associated with an increased risk of mortality (Rutledge et al., 2022). DNAm ageing scores reflecting older biological than chronological age were found to be associated with a variety of social factors, such as socioeconomic conditions (Fiorito et al., 2019; Hughes et al., 2018). However, the associations with family factors have not been comprehensively examined.

The literature on social determinants of health and DNA methylation is largely focused on old age groups or is limited in the number of age groups examined (Evans et al., 2021). Here we examine the association of partnership status with two DNAm age algorithms, ‘PhenoAge’ and ‘DunedinPACE’. Due to the processes of their derivation, we will refer to "faster" DunedinPACE and "older" PhenoAge when considering biological age that is greater than chronological age (‘accelerated' age). As the literature suggests that there may be sex interactions in the association of partnership status and health (Wang et al., 2020), we explore interactions with sex. Further, as partnership status may have different saliency in younger and older age groups (Requena & Reher, 2021), we explore interactions with age group. In addition to age and sex, a number of factors are associated with partnership and dissolution that could play a role in the association between partnership status and biological age. For example, since there is evidence on the connection between living arrangements, marital status (Grundström et al., 2021) and mental health, household composition may represent a psychosocial influence of the relationship between partnership status and biological ageing. Besides, a large body of evidence suggests that marital status is protective against poor health behaviours (Schone & Weinick, 1998).

Based on the previous evidence and the above theoretical framework, we address the following research hypotheses.

Hypothesis 1 That being partnered is associated with negative accelerated age, that is younger biological age compared to chronological age, compared to single, divorced or widowed counterparts.

Hypothesis 2 The association between partnership status and age acceleration differs between men and women. The impact of relationship disruption on men's biological age is greater than on women such that accelerated age will be apparent in unpartnered men compared to their partnered counterparts.

Hypothesis 3 The association between partnership status and age acceleration differs according to life stage. Partnership status will have a greater adverse association on older people's biological age but not for younger age groups.

## 2 Methods

### 2.1 Study design and study participants

Understanding Society, the UK Household Longitudinal Study (UKHLS), is a large, ongoing study designed to be representative of the UK population. We utilize information from the DNAm subsample from Understanding Society. Between May 2010 and July 2012 (Waves 2 and 3), participants’ blood samples were collected during nurse visits in the participants' homes, which followed, on average, 5 months after a ‘main study’ interview. Respondents were eligible for a Nurse visit if they were living in England, Scotland or Wales, adult (aged 16 or older), and met other conditions detailed in the user's guide (*Understanding Society: Waves 2 and 3 Nurse Health Assessment, 2010–2012*, 2022). All data collection methods were carried out in accordance with Information Commissioner's Office guidelines and regulations. Informed consent was obtained from all participants. The University of Essex Ethics Committee approved data collection on *Understanding Society* main study. Approval for asking for consent and the collection of biosocial data by trained nurses was obtained from the National Research Ethics Service (Understanding Society—UK Household Longitudinal Study: A Biosocial Component, Oxfordshire A REC, Reference: 10/H0604/2). The DNAm sample was restricted to participants of white ethnicity who had consented to both blood sampling and genetic analysis. We used the Illumina Methylation EPIC BeadChip which integrated over 850,000 methylation sites across the genome. After pre-processing quality control steps, including outlier removal, filtering poor-quality probes and quantile normalization, DNAm profiling data is available for 3654 participants.

**Fig. 1:**
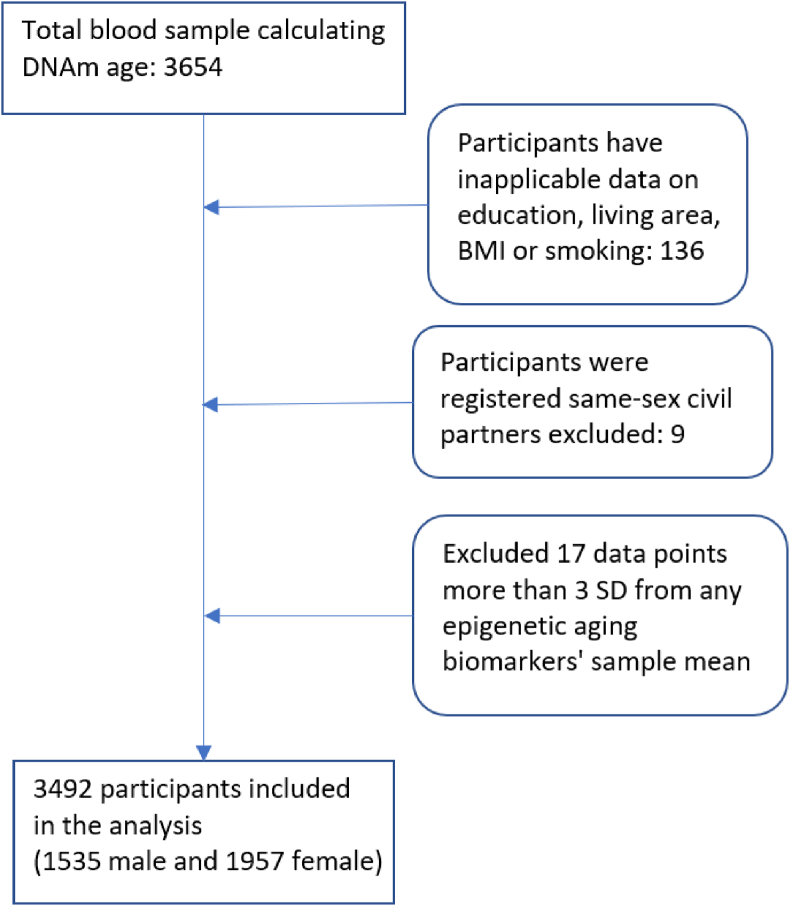
Analytical sample selection process.

After excluding inapplicable and missing data on variables, same-sex civil partners, and data points greater than 3 standard deviations (SD) from DNAm age mean values, there were 3492 participants included in the analysis (Fig. 1).

### 2.2 Measures

#### 2.2.1 Independent variable: partnership status

The exposure was self-reported current *de facto* marital status reported in the main survey, here referred to as “partnership status”. Partnership status was categorized into 4 groups, including married or living with a partner (reference), single, divorced or separated, widowed.

#### 2.2.2 Outcome variable: DNAm age acceleration

Biological ageing rates are calculated using DNAm markers ‘CpG sites’ across the genome (Bell et al., 2019). We calculated DNAm age using two DNAm algorithms: PhenoAge (Levine et al., 2018) and DunedinPACE (Belsky et al., 2022) as previously described. In UKHLS (Understanding Society: Waves 2 and 3 Nurse Health Assessment, 2010–2012, 2022), PhenoAge estimates the phenotypic age based on markers of tissue and immune function using 512 CpG sites in whole blood, and DunedinPACE captures the rate of change across 19 health-related biomarkers using 172 CpG sites in whole blood. The PhenoAge DNAm algorithm captures biological age while DunedinPACE measures the biological ‘pace of ageing', which is estimated as a measurement of an average rate of 1 year of biological ageing per year of chronological ageing. DunedinPACE, is measured in years of accelerated ageing per year of ageing. In order to measure age acceleration without the effect of chronological age, we calculated ‘PhenoAge’ DNAm age acceleration. PhenoAgeAA quantified by the difference between the amount of ageing observed based on DNAm and the amount of ageing expected based on chronological age, is calculated as the residual from regressing DNAm age (dependent variable) on chronological age (independent variable). This study includes PhenoAgeAA and DunedinPACE in the analysis. We also report the z-score standardized PhenoAgeAA and DunedinPACE, which help compare biological age metrics with "pace of ageing" metrics in descriptive analysis.

#### 2.2.3 Covariates

After a literature review (Cheung & Mui, 2022; Fiorito et al., 2019; Hughes et al., 2018; Mainous et al., 2011; Nevalainen et al., 2017; Ryan et al., 2020; Schmitz et al., 2022; Yu, 2023), several important potential covariates were identified:

*Educational attainment.* Highest educational qualification was categorized as no qualification, other qualification (entry level qualification in the UK), General Certificate of Secondary Education (GCSE) etc. (level 1 and level 2 qualifications), General Certificate of Education Advanced Level (A-level) etc. (level 3 qualifications), other higher degree (level 4 and level 5 qualifications), and degree (level 6 to 8 qualifications). *Household composition.* It was categorized into living with others vs. living alone. *Psychological distress.* The 12-item General Health Questionnaire (GHQ) was used to assess participant's psychological distress during the main survey. We used the Likert scoring method (0-1-2-3), and the scores continuously ranged from 0 to 36, with higher scores indicating greater distress. Since the GHQ score is non-normal distribution, we used the mean GHQ score (11 in our sample) as a cut-off as previously described (Shelton & Herrick, 2009) to create two groups: the absence (GHQ score <11) and the presence (GHQ score ≥11) of psychological distress. *Living area.* People's living areas were self-reported as living in urban or rural areas. *Body Mass index.* Height and weight were measured by the nurse using a portable stadiometer and digital floor scale (the Tanita BF 522 scales) (*Understanding Society: Waves 2 and 3 Nurse Health Assessment, 2010–2012*, 2022) to calculate body mass index (BMI). Adiposity was indexed by BMI which was categorized as underweight (<18.5), healthy-weight (18.5–24.9), overweight (25.0–29.9), and obese (≥30.0). *Smoking.* Smoking status was collected in the questionnaire in the main survey of wave 2 and the following three categories were created: never smoker, ex-smoker and current smoker. *Alcohol consumption.* Current drinking behaviour was assessed by questionnaire in the main survey of wave 2. As drinking 6 or more units in a single session is classed as binge drinking (Department of Health and Social Care, 2021), the participant's drinking intensity was divided into none, 0–6 units (>0 and < 6), and 6+ units ( ≥ 6).

### 2.3 Statistical analysis

We compared the bivariate associations between covariates with each exposure and outcomes, using adjusted Wald test to test overall significance. To test for sex moderation, we created three Sex × Partnership Status interaction terms, one for each partnership status group other than the reference group, and entered these as a set into the regression equation predicting DNAmAA. The association between the set of interaction terms and DNAmAA were statistically significant (P < 0.05), so all models were estimated separately by sex. Then we further tested the rationale of additionally stratifying models by age group, the p-value of the likelihood ratio test was also statistically significant.

Covariates were divided into different blocks based on the combination of existing literatures and Social Determinants of Health (SDH) conceptual framework (Solar & Irwin, 2010). This study has 5 blocks of covariates, including technical covariates (batch barcode and various cell composition estimates (Houseman et al., 2012)), demographic variables (chronological age, age^2^, educational attainment), psychosocial block (household composition and psychological distress), material block (living area), and behavioural block (adiposity, smoking, and alcohol consumption). We included the age-squared term in these analyses to account for potential nonlinearity in the association between age and DNAmAA. Because of substantial data missingness, alcohol consumption and psychological distress were examined in sensitivity analysis only.

Linear regression models were used to assess the association between partnership status and DNAmAA. Firstly, we report models stratified by sex. Model 1 estimates marital status differences in DNAmAA, adjusting for the technical covariates and demographic variables. Models 2–4 separately adds the psychosocial block (Model 2), material block (Model 3) and behavioural block (Model 4) variables to Model 1. The fully adjusted model (Model 5) examines the net association between partnership status and DNAmAA after controlling for all the covariates.

Then we report a fully adjusted linear regression model for the entire sample (changing the age from the continuous variable to the categorical variable), additionally stratified by age group (using participants' age tertiles as cut-off points). In these models, partnership status categories were further collapsed into 3 groups (married or living with a partner vs. single vs. divorced, separated, or widowed) to preserve sufficient sample size since widowed subjects were too few in the youngest age group to analyze as a separate category.

There were two different sensitivity analyses: one restricted the lower age limit of participants in order to exclude the selection effect of age on partnership status, and the other added alcohol consumption and psychological distress to the fully adjusted linear regression model. In the age-restricted analyses, we sequentially excluded those under the UK's legal minimum age (18 years) to enter a marriage (Model A1), those normally have not graduated from university in UK (≤25 years) (Model A2), and those under the mean average age of marriage at data collection time (34 years for females and 36 years for males) (Average age at marriage England and Wales, by gender | Statista) (Model A3).

All the analyses were performed with STATA 16 software.

## 3 Results

### 3.1 Description of the samples

Descriptive statistics for study variables are presented separately by partnership status groups and sex in Table 1. Among men 76.35% were partnered, 12.18% were single people, 7.88% divorced or separated, and 3.59% widowed. The corresponding figures for women were 69.90%, 9.96%, 12.47%, 7.67%. Participants had a younger mean DNAm age than chronological age in PhenoAge but not DunedinPACE. For both DNAm algorithm, the mean biological age/average pace of ageing per year for people in the single group was the youngest/slowest, while that for the widowed group was the oldest/fastest.

**Table 1:** Characteristics of the analytical sample in study of DNA methylation age (n = 3492), UK household longitudinal study.

Sex	Male					Female				
	Partnership status					Partnership status				
	N (%)/Mean (SD)					N (%)/Mean (SD)				
Variable	Total	Partnered (married or living with a partner)	Single	Divorced or separated	Widowed	Total	Partnered (married or living with a partner)	Single	Divorced or separated	Widowed
	n = 1535	n = 1172 (76.35)	n = 187 (12.18)	n = 121 (7.88)	n = 55 (3.59)	n = 1957	n = 1368 (69.90)	n = 195 (9.96)	n = 244 (12.47)	n = 150 (7.67)
Age (years)	52.68 (15.58)	53.90 (14.27)	37.63 (17.03)	56.18 (9.89)	70.23 (10.27)	51.98 (15.14)	51.84 (13.74)	36.17 (15.33)	54.37 (10.93)	69.91 (10.73)
PhenoAge (years)	45.28 (12.77)	46.09 (11.48)	33.72 (15.48)	48.60 (8.56)	59.91 (7.88)	44.85 (12.03)	44.40 (10.87)	33.56 (12.32)	47.86 (9.75)	58.71 (9.22)
PhenoAgeAA	−0.05 (5.08)	−0.12 (4.90)	−0.61 (5.33)	0.72 (5.71)	1.77 (6.04)	0.04 (5.33)	−0.31 (5.17)	0.30 (5.28)	1.30 (5.72)	0.80 (5.79)
PhenoAgeAA (z-score)	−0.01 (0.97)	−0.02 (0.94)	−0.12 (1.02)	0.14 (1.09)	0.34 (1.16)	0.01 (1.02)	−0.06 (0.99)	0.06 (1.01)	0.25 (1.10)	0.15 (1.11)
DunedinPACE	1.07 (0.14)	1.06 (0.13)	1.02 (0.14)	1.12 (0.17)	1.16 (0.12)	1.05 (0.13)	1.04 (0.13)	1.05 (0.14)	1.08 (0.13)	1.12 (0.13)
DunedinPACE (z-score)	0.60 (1.03)	0.04 (0.97)	−0.26 (1.06)	0.46 (1.25)	0.79 (0.91)	−0.05 (0.98)	−0.14 (0.95)	−0.07 (1.04)	0.20 (0.95)	0.45 (0.96)
Highest educational qualification										
Degree	367 (23.91)	300 (25.60)	38 (20.32)	23 (19.01)	6 (10.91)	391 (19.98)	301 (22.00)	40 (20.51)	38 (15.57)	12 (8.00)
Other higher degree	179 (11.66)	142 (12.12)	17 (9.09)	12 (9.92)	8 (14.55)	292 (14.92)	214 (15.64)	25 (12.82)	32 (13.11)	21 (14.00)
A-level	347 (22.61)	263 (22.44)	52 (27.81)	24 (19.83)	8 (14.55)	312 (15.94)	220 (16.08)	47 (24.10)	36 (14.75)	9 (6.00)
GCSE	327 (21.30)	238 (20.31)	47 (25.13)	26 (21.49)	16 (29.09)	470 (24.02)	319 (23.32)	59 (30.26)	64 (26.23)	28 (18.67)
Other qualification	152 (9.90)	112 (9.56)	17 (9.09)	18 (14.88)	5 (9.09)	209 (10.68)	133 (9.72)	14 (7.18)	30 (12.30)	32 (21.33)
No qualification	163 (10.62)	117 (9.98)	16 (8.56)	18 (14.88)	12 (21.82)	283 (14.46)	181 (13.23)	10 (5.13)	44 (18.03)	48 (32.00)
Household composition										
Living alone	233 (15.18)	6 (0.51)	87 (46.52)	96 (79.34)	44 (80.00)	301 (15.38)	6 (0.44)	67 (34.36)	111 (45.49)	117 (78.00)
Living with others	1304 (84.82)	1166 (99.49)	100 (53.48)	25 (20.66)	11 (20.00)	1656 (84.62)	1362 (99.56)	128 (65.64)	133 (54.51)	33 (22.00)
Living area										
Urban area	1085 (70.68)	811 (69.20)	146 (78.07)	87 (71.90)	42 (74.55)	1421 (72.61)	958 (70.03)	168 (86.15)	184 (75.41)	111 (74.00)
Rural area	450 (29.32)	361 (30.80)	41 (21.93)	34 (28.10)	14 (25.45)	536 (27.39)	410 (29.97)	27 (13.85)	60 (24.59)	39 (26.00)
Adiposity										
Healthy-weight	365 (23.78)	243 (20.73)	68 (36.36)	38 (31.40)	16 (29.09)	638 (32.60)	442 (32.31)	82 (42.05)	82 (33.61)	32 (21.33)
Underweight	8 (0.52)	2 (0.17)	6 (3.21)	0 (0.00)	0 (0.00)	24 (1.23)	11 (0.80)	8 (4.10)	2 (0.82)	3 (2.00)
Overweight	676 (44.04)	538 (45.90)	72 (38.50)	50 (41.32)	16 (29.09)	692 (35.36)	496 (36.26)	59 (30.26)	77 (31.56)	60 (40.00)
Obese	486 (31.66)	389 (33.19)	41 (21.93)	33 (27.27)	23 (41.82)	603 (30.81)	419 (30.63)	46 (23.59)	83 (34.02)	55 (36.67)
Smoking										
Never smoker	727 (47.36)	551 (47.01)	104 (55.61)	50 (41.32)	22 (40.00)	1085 (55.44)	798 (58.33)	103 (52.82)	104 (42.62)	80 (53.33)
Ex-smoker	509 (33.16)	422 (36.01)	32 (17.11)	31 (25.62)	24 (43.64)	503 (25.70)	359 (26.24)	27 (13.85)	71 (29.10)	46 (30.67)
Current smoker	299 (19.48)	199 (16.98)	51 (27.27)	40 (33.06)	9 (16.36)	369 (18.86)	211 (15.42)	65 (33.33)	69 (28.28)	24 (16.00)

DNAm: DNAm methylation.

Adiposity was indexed by BMI which was categorized as underweight (<18.5), healthy-weight (18.5–24.9), overweight (25.0–29.9), and obese (≥30.0).

### 3.2 Association of DNAmAA with partnership status by sex

We first evaluated multivariate association between partnership status and DNAmAA after statistically adjusting for covariates and stratifying by sex (Table 2). From Model 1 to Model 3, compared to being partnered, being single was significantly associated with positive DunedinPACE for females; being widowed showed significantly greater positive DNAmAA for both algorithms; and being divorced or separated was significantly associated with positive PhenoAgeAA for females and positive DunedinPACE in men and women. Positive DunedinPACE in single females was not robust to adjustment for behavioural variables (model 4). When adjusting for all covariates (Model 5), widows were epigenetically “older" in comparison with partnered people for both algorithms. Widows’ PhenoAgeAA was 2.00 years older (95% CI = 0.46, 3.54) for men and 1.29 years older (95% CI = 0.25, 2.33) for women; DunedinPACE was 0.05 biological years per chronological year faster (95% CI = 0.01, 0.08) for men and 0.03 biological years per chronological year faster (95% CI = 0.01, 0.05) for women. Positive age acceleration is apparent in all groups in final models in comparison to partnered groups, however, these findings were not all significant.

**Table 2:** Regression models of the association between partnership status and DNAm AA, stratified by sex (males n = 1535, females n = 1957).

	Model 1^a^		Model 2^b^		Model 3^c^		Model 4^d^		Model 5^e^	
	Male	Female	Male	Female	Male	Female	Male	Female	Male	Female
	Coeff. (95% CI)	Coeff. (95% CI)	Coeff. (95% CI)	Coeff. (95% CI)	Coeff. (95% CI)	Coeff. (95% CI)	Coeff. (95% CI)	Coeff. (95% CI)	Coeff. (95% CI)	Coeff. (95% CI)
Panel A: PhenoAgeAA										
Partnership status										
Partnered (married or living with a partner)	Reference									
Single	0.31 (−0.51 to 1.13)	0.55 (−0.25 to 1.35)	0.30 (−0.72 to 1.32)	0.35 (−0.52 to 1.23)	0.30 (−0.52 to 1.12)	0.50 (−0.30 to 1.30)	0.38 (−0.42 to 1.18)	0.32 (−0.46 to 1.11)	0.29 (−0.71 to 1.29)	0.08 (−0.79 to 0.94)
Divorced or separated	0.27 (−0.62 to 1.16)	1.14 (0.48–1.79)***	0.26 (−0.95 to 1.47)	0.93 (0.18–1.69)*	0.25 (−0.63 to 1.14)	1.11 (0.45–1.77)***	0.25 (−0.62 to 1.12)	0.86 (0.21–1.51)**	0.12 (−1.07 to 1.31)	0.63 (−0.11 to 1.37)
Widowed	2.23 (0.90–3.56)***	1.89 (1.00–2.78)***	2.22 (0.65–3.80)**	1.57 (0.51–2.63)**	2.19 (0.86–3.52)***	1.85 (0.96–2.75)***	2.15 (0.85–3.45)***	1.65 (0.77–2.52)***	2.00 (0.46–3.54)*	1.29 (0.25–2.33)*
R-squared	0.193	0.232	0.193	0.232	0.195	0.233	0.236	0.268	0.237	0.269
Adjusted R-squared	0.163	0.210	0.162	0.209	0.164	0.210	0.205	0.244	0.204	0.245
Panel B: DunedinPACE										
Partnership status										
Partnered (married or living with a partner)	Reference									
Single	0.01 (−0.01 to 0.03)	0.02 (0.00–0.04)*	0.03 (0.00–0.05)*	0.03 (0.01–0.05)*	0.01 (−0.01 to 0.03)	0.02 (0.00–0.04)*	0.01 (−0.01 to 0.03)	0.01 (−0.01 to 0.03)	0.02 (−0.00 to 0.05)	0.01 (−0.01 to 0.03)
Divorced or separated	0.04 (0.01–0.06)***	0.03 (0.01–0.05)***	0.06 (0.03–0.09)***	0.03 (0.01–0.05)***	0.04 (0.01–0.06)***	0.03 (0.01–0.05)***	0.03 (0.01–0.05)**	0.02 (0.00–0.03)*	0.04 (0.02–0.07)**	0.02 (0.00–0.03)*
Widowed	0.04 (0.01–0.08)*	0.04 (0.02–0.06)***	0.06 (0.02–0.10)***	0.05 (0.02–0.07)***	0.04 (0.01–0.07)*	0.04 (0.02–0.06)***	0.03 (0.00–0.06)*	0.03 (0.01–0.05)**	0.05 (0.01–0.08)**	0.03 (0.01–0.05)*
R-squared	0.322	0.228	0.342	0.228	0.326	0.230	0.455	0.402	0.457	0.403
Adjusted R-squared	0.297	0.205	0.298	0.205	0.299	0.207	0.432	0.383	0.434	0.383

CI: Confidence interval.

Coef.: Coefficient.

⁎ p < 0.05, ⁎⁎ p < 0.01, ⁎⁎⁎ p < 0.001.

a Adjusted for technical covariates (barcode and various cell composition estimates), demographic variables (chronological age, age2, education).

b Adjusted for technical covariates, demographic variables, psychosocial block (household composition).

c Adjusted for technical covariates, demographic variables, material block (living area).

d Adjusted for technical covariates, demographic variables, behavioural block (adiposity, smoking).

e Adjusted for technical covariates, demographic variables, psychosocial block, material block, behavioural block.

### 3.3 Results when considering the age moderation effect

Next, we evaluated whether the association between marital status and positive age acceleration changed when further stratified by age groups (Table 3). In younger age groups, by the PhenoAge algorithm, single men were epigenetically "younger" than chronological age (PhenoAgeAA was 1.96 years younger), and by DunedinPACE algorithm, younger single men were ageing epigenetically slower (DunedinPACE was 0.03 biological years per chronological year slower) compared to partnered counterparts in the same age group. Singlehood was significantly associated with greater epigenetic ageing (4.27 years older) among older men, especially using the PhenoAge algorithm. Divorced, separated, or widowed status was associated with greater epigenetic age in younger (DunedinPACE was 0.09 biological years per chronological year faster) and older (DunedinPACE was 0.07 biological years per chronological year faster) men compared to being partnered.

**Table 3:** Results of linear regressions using DNAm AA as outcomes, stratified by age group and sex.

	Male				Female			
	Full sample^a^ (n = 1535)	16–44 years^b^	45–60 years^b^	61 years and older^b^	Full sample^a^ (n = 1957)	16–44 years^b^	45–60 years^b^	61 years and older^b^
	Coeff. (95% CI)	Coeff. (95% CI)	Coeff. (95% CI)	Coeff. (95% CI)	Coeff. (95% CI)	Coeff. (95% CI)	Coeff. (95% CI)	Coeff. (95% CI)
Panel A: PhenoAgeAA								
Partnership status								
Partnered (married or living with a partner)	Reference				Reference			
Single	−0.68 (−1.60 to 0.24)	−1.96 (-3.01 to -0.91)***	1.66 (−0.39 to 3.71)	4.27 (0.94 to 7.60)**	−0.23 (−1.04 to 0.58)	−0.24 (−1.20 to 0.72)	1.24 (−0.59 to 3.08)	−1.90 (−4.88 to 1.08)
Divorced, separated, or widowed	0.19 (−0.92 to 1.30)	1.13 (−1.20 to 3.46)	0.80 (−1.07 to 2.66)	2.34 (−032 to 4.99)	0.65 (−0.05 to 1.35)	−0.13 (−1.52 to 1.26)	0.56 (−0.48 to 1.59)	1.26 (−0.63 to 3.15)
Panel B: DunedinPACE								
Partnership status								
Partnered (married or living with a partner)	Reference				Reference			
Single	−0.01 (−0.03 to 0.01)	−0.03 (−0.06 to −0.01)*	0.05 (0.00–0.09)*	0.07 (−0.01 to 0.14)	0.01 (−0.01 to 0.03)	0.01 (−0.01 to 0.03)	0.02 (−0.02 to 0.06)	0.01 (−0.05 to 0.07)
Divorced, separated, or widowed	0.03 (0.00–0.05)*	0.09 (0.03–0.14)**	0.04 (−0.01 to 0.08)	0.07 (0.02–0.13)*	0.02 (0.01–0.04)**	0.00 (−0.03 to 0.04)	0.02 (−0.00 to 0.04)	0.03 (−0.01 to 0.07)

⁎ p < 0.05, ⁎⁎ p < 0.01, ⁎⁎⁎ p < 0.001.

a Adjusted for technical covariates, age categories, education, household composition, living area, adiposity, smoking.

b Adjusted for technical covariates, education, household composition, living area, adiposity, smoking.

### 3.4 Sensitivity analysis

For sensitivity analysis (Table Appendix A), when excluding people aged less than 18, less than 25, and less than UK average age at marriage, widows still exhibited positive DNAmAA in comparison with partnered people, using both algorithms. Those divorced or separated males were ageing significantly faster than chronological age compared to partnered counterparts when using DunedinPACE algorithm. Among those beyond the mean age at marriage in UK, the values for single males measured by both algorithms became statistically significant, with PhenoAgeAA 1.89 years older (95% CI = 0.43, 3.35) and DunedinPACE 0.05 biological years per chronological year faster (95% CI = 0.02, 0.08) compared to partnered counterparts.

The addition of alcohol consumption and psychological distress to the model reduced sample size (n = 3021) and hence power but did not affect conclusions in DunedinPACE for females (Table Appendix B). When adding two more covariates to fully adjusted linear regression models, compared with partnered participants, DunedinPACE for divorced/separated and widowed women was 0.02 (95% CI = 0.01, 0.04) and 0.03 (95% CI = 0.01, 0.06) biological years per chronological year faster, and PhenoAgeAA for divorced/separated and widowed women was 1.09 (95% CI = 0.28, 1.90) and 1.63 (95% CI = 0.49, 2.76) years older, respectively. The standardized regression coefficients shows that partnership status has a strong association with biological ageing.

## 4 Discussion

We demonstrate that partnership status is associated with DNAm age acceleration, such that both men and women who are widowed and divorced are biologically older than chronological age to a greater extent than their counterparts in partnerships. We observe an interaction with sex and age; for example, being single for men was a protective factor against ageing at a younger age, but remaining single was associated with worse biological ageing with increasing age for both women and men. Our results accord with previous observations that being in a relationship, including marriage or cohabitation, confers a health advantage (Crowley, 2019; Liu & Waite, 2014; McFarland et al., 2013; Yu, 2023).

We observe that divorced, separated, or widowed people age faster, when assessed with DNA methylation, compared to counterparts in partnerships, which is consistent with Hypothesis 1. Our observations accord with studies that suggest that divorce and widowhood are associated with an increased risk of poor health (Tatangelo et al., 2017). Such findings may be explained by the stress model, which states that relationship disruption may serve as a psychosocial stressor, and psychosocial stress may be associated with DNAm changes (Palma-Gudiel et al., 2015; Schiele et al., 2020). Our finding that both single older men and women were biologically older than their counterparts accord with the resource model, which proposes that being single may represent a lack of material and social resources, with a resultant adverse association with health.

Our findings do not fully accord with a recent study which found greater epigenetic ageing among single women but not for single men in the Health and Retirement Study (HRS) (Yu, 2023). Here, consistent with hypothesis 2, we found that the association between partnership status and DNAm age acceleration is more pronounced in men than women, especially using DunedinPACE algorithm. Such findings may be explained by previous research on observed patterns in sex differences in social network composition, in which men typically receive more emotional support and favourable regulation of health behaviours from family than women do (Carr & Springer, 2010; McKenzie et al., 2018; Williams & Umberson, 2004), thus men's health status may be more influenced by partnership status.

Age-related changes in biology can occur before middle age, however, the study of marital status and epigenetic markers of age has been restricted to older age groups. Studies have examined the associations between partnership status and health conditions in younger age groups, but less using DNAm as an ageing biomarker like us. Interestingly, Henning-Smith and Gonzales (2020) who also divided participants to younger adults, middle-aged adults, and older adults, described a similar pattern of association by age of marital status and self-rated health as we observed with ‘PhenoAge’ (Henning-Smith & Gonzales, 2020). The literature on additional molecular biomarkers of ageing, such as telomere length and attrition (Mainous et al., 2011; Chen et al., 2020; Whisman et al., 2016; Yu & Liu, 2021) are also limited to middle and older age groups. For example Mainous (2011) described the association between marital status and telomere length in groups aged 40–64, and reported shorter telomere length in unmarried groups independently of social support and health behaviours (Mainous et al., 2011). Therefore, it is recommended that further studies explore the impact of partnership status on DNA methylation algorithms and other biological ageing measurements from young adults and for the entire age groups. In addition, there have been secular changes in the rates of marriage, informal partnerships, divorce and age of first marriage. Studies that have restricted age groups are likely not be able to observe the impacts of these changes. Thus, our study is likely to capture this greater rate of informal partnership in younger or middle-aged participants not apparent in studies that are restricted to older age groups. Indeed, we observe that our sample has more people in partnerships in middle age, while we observe the greatest rate of widowhood in our older age groups.

We found single status is sensitive to ageing and differs between men and women, which is partially consistent with Hypothesis 2 and 3. Our study found that at a younger age, partnered men were biologically older than non-partnered counterparts. This finding suggests that "adverse selection" may be at play in young men, i.e. less healthy people may be more inclined to enter into marriage, which is consistent with previous research (Mr & Sloggett, 1998). This contradicts the literature on “positive selection” (Murray, 2000), which suggests that more healthy people enter into partnerships.

Our finding that partnered older age men are biologically younger may provide insight into the association of partnership dissolution and biological age as it is likely that those enter an early relationship may also be more likely to also dissolute relationships more readily. An alternative explanation is there is reverse causation such that those in poor health may not marry in middle age. We couldn't address selection out of marriage directly as we conducted a cross sectional analysis. Although our age specific analyses suggest that unmarried younger men are healthier than married counterparts arguing for "adverse selection" explanation, these may represent cohort specific effects and therefore may not provide insight into mechanisms that may be in play for older age groups. For example, we did not know whether healthy people may be able to opt out of marriage more freely, which we have not accounted for in our analyses. For all DNAm algorithms, we found being widowed was associated with DNAmAA in both sexes. At a population level, given that women generally have high probability of outliving men (Zarulli et al., 2018), they may be more likely than men to become widowed and remain widowed longer in their later lives.

A number of potential pathways are proposed in our study, including material, psychosocial and behavioural pathways. The biomarkers of ageing employed in this work are associated with a number of factors. Many associations with covariates have been reported previously, for example disadvantaged SEP and positive accelerated age (Bao et al., 2022; Evans et al., 2021; George et al., 2021; Hughes et al., 2018), but others, such as the positive accelerated age we observe in groups living in urban areas, which have not been described previously (Evans et al., 2021) may require further investigation.

We infer that biological ageing processes underly some psychosocial, material, and behavioural mechanisms, as we see associations between partnership status and DNAm independently and with a number of adjustments. We did not see an association between our ageing biomarkers and psychological distress, but this may be because our instrument, the GHQ, may not be sensitive to the measures of mental health pertinent to marital status or that we have not examined the psychosocial environment more broadly, for example marital relationship quality, which is associated with health (Kiecolt-Glaser & Wilson, 2017). Although this study controlled for household composition and psychological distress as psychosocial factors, future research should consider other stress-related psychosocial factors. For example, stress biomarkers such as cortisol (Adam & Kumari, 2009), inflammatory markers (Marsland et al., 2017) or allostatic load (Guidi et al., 2021) could be controlled in order to investigate whether the association between partnership status and epigenetic ageing rate is mediated through stress-related pathways.

This study had several strengths as it is based on a national study, it comprised a large sample with representation from almost the entire adult age range. We examined more than 1 s generation algorithm. However, the sample was restricted to those reporting white/European ethnicity, and therefore results may not be generalizable to other ethnic groups. Measurement of DNAm was made on one occasion in adults and therefore it is not possible to examine whether age acceleration occurred before divorce or widowhood or vice versa and further it is not possible to assess within-person change in adulthood. We did not examine quality of marital relationships, which are also associated with health (Kiecolt-Glaser & Wilson, 2017) and may precede marital dissolution. Our analyses suggest that psychological distress, which may be associated with poor marital relationships, divorce or widowhood (Foran et al., 2015) did not play a role in the associations examined. However, there was a five-month delay between the collection of mental health data and blood sample collection and we do not know the stability or not of the DNAmethylation algorithms. The material, psychosocial and behavioural factors examined in this study may be mediators rather than moderators or confounders and thus there may be overadjustment. We examined two algorithms - there are additional algorithms that have been developed and can be tested. We did not adjust for multiple testing, as the strength of associations indicated that our main conclusions would not be altered by a Bonferroni correction. In stratified analyses, some numbers were small and therefore it is possible that these analyses are underpowered. The algorithms employed in this analysis have been tested and trained against biomarkers that vary with age and may therefore reflect the processes that they capture. The variety of associations apparent for these algorithms in this and additional studies (Bao et al., 2022; Raffington & Belsky, 2022; Thomas et al., 2023) suggests that our analyses may be subject to residual confounding. A focus on estimators of age restricts analyses to a small subset of methylation sites across the genome and thus limits an analysis of partnership status with DNA methylation more broadly. Broader analyses have the potential to uncover wider pathways by which social factors and health might be associated.

## 5 Conclusions

We conclude that the health disadvantage associated with divorce/separation and widowhood is apparent when examining biomarkers of ageing measured with DNA methylation across the adult age span. For single people, there is heterogeneity in our observations such that age and sex specific associations are apparent. The findings highlight the importance of partnership in people's ageing process, and these findings will be helpful for health policymakers and practitioners who seek to better design effective interventions targeted at vulnerable subpopulations and life stages to minimize inequalities in the biological ageing process.

## Ethical statement

All methods were carried out in accordance with Information Commissioner's Office guidelines and regulations. Informed consent was obtained from all participants. The University of Essex Ethics Committee approved data collection on *Understanding Society* main study. Approval for asking for consent and the collection of biosocial data by trained nurses was obtained from the National Research Ethics Service (Understanding Society—UK Household Longitudinal Study: A Biosocial Component, Oxfordshire A REC, Reference: 10/H0604/2).

## Funding

DNA methylation measurement in UKHLS was funded through enhancements to the 10.13039/501100000269Economic and Social Research Council (10.13039/501100000269ESRC) Grants ES/K005146/1 and ES/N00812X/1. WW and AD are Soc-B students (10.13039/501100000269ESRC ES/T00200X/1, project reference numbers: 2765592 and 2765580 respectively). Y.B. was partially supported by the 10.13039/501100000269ESRC (ES/N00812X/1). M.K. is supported by the 10.13039/100010046University of Essex, 10.13039/501100000269ESRC (RES-596-28-0001) and 10.13039/501100000269ESRC (ES/S012486/1; ES/T014083/1).

## CRediT authorship contribution statement

Wen Wang: Formal analysis, investigation, writing – original draft, writing – review & editing.

Anna Dearman: Writing – review & editing.

Yanchun Bao: conceptualisation, investigation, writing – review & editing.

Meena Kumari: conceptualisation, supervision, writing – review & editing.

## Declaration of competing interest

The authors declare no competing interests.

## Figures and Tables

**Table:** 

	Sensitivity analyses A1^a^		Sensitivity analyses A2^b^		Sensitivity analyses A3^c^	
	Male	Female	Male	Female	Male	Female
	Coeff. (95% CI)	Coeff. (95% CI)	Coeff. (95% CI)	Coeff. (95% CI)	Coeff. (95% CI)	Coeff. (95% CI)
Panel A: PhenoAgeAA						
Partnership status						
Partnered (married or living with a partner)	Reference					
Single	0.38 (−0.64 to1.40)	0.18 (−0.69 to 1.06)	0.91 (−0.30 to 2.12)	0.01 (−0.95 to 0.96)	1.89 (0.43 to 3.35)*	−0.15 (−1.32 to 1.01)
Divorced or separated	0.19 (−1.01 to 1.39)	0.67 (−0.08 to 1.41)	0.54 (−0.75 to 1.83)	0.62 (−0.14 to 1.38)	0.93 (−0.49 to 2.35)	0.65 (−0.15 to 1.45)
Widowed	2.04 (0.49–3.58)**	1.31 (0.27–2.35)*	2.31 (0.71–3.92)**	1.22 (0.16–2.27)*	2.60 (0.89–4.32)**	1.14 (0.04–2.24)*
Panel B: DunedinPACE						
Partnership status						
Partnered (married or living with a partner)	Reference					
Single	0.02 (0.00–0.05)*	0.01 (−0.01 to 0.03)	0.03 (0.00–0.06)*	0.02 (−0.01 to 0.04)	0.05 (0.02–0.08)**	0.01 (−0.01 to 0.04)
Divorced or separated	0.04 (0.02–0.07)***	0.02 (0.00–0.03)*	0.05 (0.02–0.08)**	0.02 (0.00–0.03)*	0.06 (0.03–0.09)***	0.02 (−0.01 to 0.03)
Widowed	0.05 (0.01–0.08)**	0.03 (0.01–0.05)*	0.05 (0.01–0.09)**	0.03 (0.00–0.05)*	0.06 (0.02–0.10)**	0.02 (−0.01 to 0.05)

Adjusted for technical covariates, demographic variables, psychosocial block, material block, behavioural block.

⁎ p < 0.05, ⁎⁎ p < 0.01, ⁎⁎⁎ p < 0.001.

a Excluded those under the legal minimum age (18 years) to enter into a marriage, n = 3460.

b Keep those beyond 25 years old, n = 3334.

c Keep those beyond the mean average age at marriage (34 years for female and 36 years for male), n = 2955.

**Table:** 

	PhenoAgeAA		z_PhenoAgeAA†		DunedinPACE		z_DunedinPACE†	
	Male	Female	Male	Female	Male	Female	Male	Female
	Coeff. (95% CI)	Coeff. (95% CI)	Beta (95% CI)	Beta (95% CI)	Coeff. (95% CI)	Coeff. (95% CI)	Beta (95% CI)	Beta (95% CI)
Partnership status								
Partnered (married or living with a partner)				Reference				
Single	0.32 (−0.78 to 1.43)	0.33 (−0.62 to 1.28)	0.06 (−0.15 to 0.27)	0.06 (−0.12 to 0.24)	0.01 (−0.01 to 0.04)	0.01 (−0.01 to 0.03)	0.10 (−0.09 to 0.28)	0.09 (−0.07 to 0.24)
Divorced or separated	0.47 (−0.83 to 1.78)	1.09 (0.28 to 1.90)**	0.09 (−0.16 to 0.34)	0.21 (0.05 to 0.36)**	0.04 (0.02 to 0.07)**	0.02 (0.01 to 0.04)*	0.33 (0.11 to 0.55)**	0.15 (0.01 to 0.28)*
Widowed	1.78 (0.10–3.47)*	1.63 (0.49–2.76)**	0.34 (0.02–0.66)*	0.31 (0.09–0.53)**	0.03 (−0.01 to 0.07)	0.03 (0.01–0.06)**	0.22 (−0.06 to 0.51)	0.26 (0.07–0.45)**
Age	0.33 (0.22–0.44)***	0.23 (0.13–0.33)***	0.96 (0.64–1.28)***	0.67 (0.37–0.96)***	0.01 (0.00–0.01)***	−0.01 (−0.01 to 0.00)	0.65 (0.37–0.93)***	−0.02 (−0.27 to 0.23)
Highest educational qualification								
No qualification				Reference				
Other qualification	−0.76 (−1.90 to 0.38)	−0.25 (−1.20 to 0.69)	−0.14 (−0.36 to 0.07)	−0.05 (−0.23 to 0.13)	−0.04 (−0.07 to −0.01)**	−0.02 (−0.04 to −0.01)*	−0.29 (−0.49 to −0.10)**	−0.16 (−0.32 to −0.01)*
GCSE	−0.74 (−1.72 to 0.25)	−0.97 (−1.79 to −0.15)*	−0.14 (−0.33 to 0.05)	−0.19 (−0.34 to −0.03)*	−0.06 (−0.08 to −0.03)***	−0.03 (−0.05 to −0.01)***	−0.42 (−0.58 to −0.25)***	−0.23 (−0.36 to −0.09)***
A-level	−0.80 (−1.77 to 0.16)	−1.25 (−2.16 to −0.35)**	−0.15 (−0.34 to 0.03)	−0.24 (−0.41 to −0.07)**	−0.05 (−0.08 to −0.03)***	−0.03 (−0.05 to −0.01)**	−0.40 (−0.57 to −0.24)***	−0.24 (−0.39 to −0.09)**
Other higher degree	−0.95 (−2.04 to 0.14)	−1.24 (−2.14 to −0.34)**	−0.18 (−0.39 to 0.03)	−0.24 (−0.41 to −0.07)**	−0.06 (−0.08 to −0.03)***	−0.04 (−0.06 to −0.02)***	−0.42 (−0.60 to −0.23)***	−0.30 (−0.45 to −0.15)***
Degree	−0.62 (−1.61 to 0.38)	−1.05 (−1.95 to −0.15)*	−0.12 (−0.31 to 0.07)	−0.20 (−0.37 to −0.03)*	−0.07 (−0.09 to −0.05)***	−0.05 (−0.07 to −0.03)***	−0.52 (−0.69 to −0.35)***	−0.35 (−0.50 to −0.20)***
Household composition								
Living alone				Reference				
Living with others	0.17 (−0.99 to 1.34)	−0.05 (−0.94 to 0.84)	0.03 (−0.19 to 0.26)	−0.01 (−0.18 to 0.16)	0.01 (−0.01 to 0.04)	0.01 (−0.01 to 0.03)	0.11 (−0.09 to 0.30)	0.05 (−0.09 to 0.20)
Living area								
Urban area				Reference				
Rural area	−0.21 (−0.76 to 0.34)	−0.14 (−0.65 to 0.37)	−0.04 (−0.14 to 0.06)	−0.03 (−0.13 to 0.07)	−0.01 (−0.02 to 0.00)	−0.01 (−0.02 to 0.01)	−0.07 (−0.16 to 0.02)	−0.06 (−0.15 to 0.02)
Smoking								
Never smoker				Reference				
Ex-smoker	1.08 (0.49–1.66)***	0.46 (−0.08 to 1.00)	0.21 (0.09–0.32)***	0.09 (−0.02 to 0.19)	0.03 (0.01–0.04)***	0.04 (0.03–0.05)***	0.21 (0.11–0.31)***	0.28 (0.19–0.37)***
Current smoker	2.37 (1.66–3.08)***	2.14 (1.49–2.78)***	0.45 (0.32–0.59)***	0.41 (0.29–0.53)***	0.12 (0.11–0.14)***	0.13 (0.11–0.14)***	0.93 (0.81–1.05)***	0.94 (0.83–1.04)***
**Adiposity**								
Healthy-weight				Reference				
Underweight	0.47 (−3.05 to 3.99)	−1.54 (−3.71 to 0.62)	0.09 (−0.58 to 0.76)	−0.30 (−0.71 to 0.12)	−0.02 (−0.10 to 0.06)	−0.05 (−0.10 to 0.00)	−0.17 (−0.77 to 0.42)	−0.36 (−0.71 to 0.01)
Overweight	1.13 (0.47–1.78)***	0.01 (−0.54 to 0.56)	0.22 (0.09–0.34)***	0.01 (−0.10 to 0.11)	0.02 (0.01–0.04)**	0.02 (0.01–0.04)***	0.17 (0.06–0.28)**	0.18 (0.09–0.27)***
Obese	1.85 (1.15–2.55)***	1.38 (0.80–1.96)***	0.35 (0.22–0.49)***	0.26 (0.15–0.38)***	0.06 (0.05–0.08)***	0.08 (0.07–0.09)***	0.46 (0.34–0.58)***	0.60 (0.51–0.70)***
Alcohol (drinking intensity in a single session)								
None				Reference				
<6 units	−0.24 (−1.00 to 0.52)	−0.07 (−0.62 to 0.49)	−0.05 (−0.19 to 0.10)	−0.01 (−0.12 to 0.09)	−0.01 (−0.03 to 0.01)	−0.01 (−0.01 to 0.01)	−0.07 (−0.20 to 0.06)	−0.02 (−0.11 to 0.07)
6 units and more	0.35 (−0.34 to 1.04)	0.36 (−0.22 to 0.95)	0.07 (−0.06 to 0.20)	0.07 (−0.04 to 0.18)	−0.01 (−0.02 to 0.01)	−0.01 (−0.02 to 0.01)	−0.06 (−0.18 to 0.06)	−0.04 (−0.14 to 0.06)
Psychological distress								
Absence of psychological distress				Reference				
Presence of psychological distress	0.22 (−0.30 to 0.74)	0.14 (−0.32 to 0.60)	0.04 (−0.06 to 0.14)	0.03 (−0.06 to 0.11)	0.01 (−0.01 to 0.02)	0.01 (−0.01 to 0.02)	0.06 (−0.03 to 0.15)	0.05 (−0.02 to 0.13)

⁎ p < 0.05, ⁎⁎ p < 0.01, ⁎⁎⁎ p < 0.001.

†For easier comparison, the effect-sizes are reported as standardized regression coefficients.

## Data Availability

All data are available from the UKDA. UK Data Service. SN:7251. 10.5255/UKDA-SN-7251-3
